# Feasibility, Acceptability, and Outcomes of Project Rally: Pilot Study of a YMCA-Based Pickleball Program for Cancer Survivors

**DOI:** 10.3390/healthcare13030256

**Published:** 2025-01-28

**Authors:** Nathan H. Parker, Alexandre de Cerqueira Santos, Riley Mintrone, Kea Turner, Steven K. Sutton, Tracey O’Connor, Jeffrey Huang, Morgan Lael, Summer Cruff, Kari Grassia, Mart Theodore De Vera, Morgan Bean, Rachel Carmella, Susan T. Vadaparampil, Jennifer I. Vidrine

**Affiliations:** 1 Department of Health Outcomes and Behavior, Moffitt Cancer Center, Tampa, FL 33612, USA; 2 YMCA of the Suncoast, Clearwater, FL 33763, USA; 3 Division of Health Systems, Policy, and Innovations, School of Nursing, University of North Carolina at Chapel Hill, Chapel Hill, NC 27599, USA; 4 Lineberger Comprehensive Cancer Center, University of North Carolina at Chapel Hill, Chapel Hill, NC 27599, USA; 5 Department of Biostatistics and Bioinformatics, Moffitt Cancer Center, Tampa, FL 33612, USA; 6 Department of Breast Oncology, Moffitt Cancer Center, Tampa, FL 33612, USA; 7 Department of Anesthesiology, Moffitt Cancer Center, Tampa, FL 33612, USA; 8 Flatiron Health, New York, NY 10013, USA; 9 Office of Community Outreach, Engagement, & Equity, Moffitt Cancer Center, Tampa, FL 33612, USA

**Keywords:** exercise, sports, survivorship, community support

## Abstract

Background: Physical activity helps cancer survivors ameliorate physiological and psychosocial effects of disease and treatments. However, few cancer survivors meet physical activity recommendations, with many facing barriers such as limited interest, enjoyment, and social support. It is critical to develop enjoyable and supportive physical activity programs to improve well-being among the growing population of cancer survivors. Pickleball is increasingly popular due to its unique combination of physical activity, friendly competition, and social interaction, making it a promising strategy to increase and sustain physical activity in cancer survivorship. Objective: We examined feasibility, acceptability, and preliminary outcomes in a single-arm pilot study of Project Rally, a YMCA-based pickleball program for adult cancer survivors. Results: Twenty-one cancer survivors and seven family or friend partners enrolled in Project Rally with a targeted program duration of 3–7 months. All programming and study assessments occurred at a single YMCA with coaching and supervision from a YMCA exercise trainer and certified pickleball coach. Feasibility and acceptability were strong and met a priori targets for recruitment, retention, intervention adherence, and ratings of program aspects. Participants demonstrated significant increases in physical activity and improvements in aspects of fitness, physical functioning, and social support. Conclusion: These results will inform further development of the Project Rally program to increase physical activity and improve cancer survivorship outcomes, including efforts to expand the program’s scale and reach more survivors via community-based delivery.

## 1. Introduction

The National Cancer Institute defines cancer survivorship as the period from diagnosis through the rest of life [1]. With an aging population and advancements in early detection and treatment, the number of cancer survivors in the United States is anticipated to increase from 18.1 million to 26.0 million (nearly 44%) between 2022 and 2040 [2]. This creates an increasingly critical need to develop programs that increase physical activity (PA) among cancer survivors. PA promotion is an important component of cancer survivorship care and research [3], because it can help mitigate the adverse effects of cancer and cancer therapies while empowering survivors to lead fulfilling lives. PA provides numerous benefits in cancer survivorship, including improvements in cardiorespiratory fitness, physical functioning, body composition, psychological stress, and health-related quality of life [4,5].

Despite the widespread adoption of PA guidelines for cancer survivors, an estimated 53–83% do not achieve recommendations [6,7]. Intra- and interpersonal barriers to PA adoption and maintenance among cancer survivors include inadequate social support and lack of enjoyment [8,9]. Targeting these barriers in program design may help increase PA uptake and maintenance among cancer survivors and proliferate its physiological and psychosocial benefits. PA programs that have focused on increasing social support and enjoyment among cancer survivors, such as by incorporating group exercise or participation in sports or active games, have demonstrated promising improvements in PA and related benefits [10,11,12].

Pickleball has broad appeal, and its 220% growth in regular players since 2020 makes it the fastest growing sport in the United States [13]. Wide accessibility, social interaction, and friendly competition have helped motivate beginners of all ages and abilities to become regular pickleball players. Much of the game’s growth has been among older adults, with those 65 or older comprising nearly a third of frequent players [13]. A recent study involving pickleball among older adults demonstrated a substantial increase in moderate-to-vigorous PA (MVPA), with participants accumulating an extra 68 min of MVPA during days on which they played [14]. Middle-aged and older adult pickleball players have demonstrated improvements in fitness, cardiometabolic risk profiles, and cognitive functioning [15,16]. These outcomes would be particularly beneficial for cancer survivors, among whom the detrimental physiological and psychosocial impacts of disease and treatments frequently persist and increase risk for chronic health challenges [4]. However, the feasibility and acceptability of pickleball programming among cancer survivors have not been established.

Pickleball also provides an important opportunity to design, deliver, and evaluate a community-based PA program for cancer survivors. Partnering with community organizations, such as the YMCA, helps overcome implementation barriers to PA programming. For example, YMCA locations are widely accessible, with over 2500 locations across 10,000 communities. Widespread YMCA implementation of effective PA programs can increase access for a large and diverse population in need of survivorship care. YMCAs commonly serve communities with limited economic resources, such as by offering financial assistance for membership. Furthermore, the YMCA has successfully scaled up and sustained evidence-based exercise programs (e.g., LIVESTRONG^®^ at the YMCA), making it an ideal partner for intervention delivery [17,18]. Despite calls to prioritize community-based delivery of PA interventions for cancer survivors to enhance generalizability, implementation and evaluation of PA interventions in community settings have been limited [19].

Pickleball has potential to increase PA and improve related outcomes in cancer survivorship, and community-based delivery may accelerate scaling up for widespread reach. However, it is important to establish the feasibility and acceptability of pickleball programming at a smaller scale before expanding implementation. The primary objective of this study was to evaluate the feasibility and acceptability of Project Rally, a YMCA-based pickleball program for cancer survivors, in a single-arm pilot study. Project Rally represents a research partnership between Moffitt Cancer Center (MCC), a National Cancer Institute-designated comprehensive cancer center in Tampa, Florida, and the YMCA of the Suncoast, a non-profit community organization operating in the MCC catchment area. We also aimed to evaluate preliminary outcomes from Project Rally including PA, social support, PA enjoyment, stress, health-related quality of life, physical functioning, and functional fitness.

## 2. Materials and Methods

This study involved a single-group pilot investigation of the Project Rally program with pre- and post-program assessments. Project Rally opened in September 2023 and enrolled participants on a rolling basis until January 2024 with the aim of engaging participants in 3–7 months of programming. Participants underwent a baseline assessment upon study enrollment, and all follow-up assessments were conducted in April 2024.

### 2.1. Participants

Cancer survivors and their family or friend partners were eligible to participate in the Project Rally pilot study. To be eligible, cancer survivors had to (1) be at least 18 years old, (2) be able to speak and read English, (3) have been diagnosed with any type of cancer excluding a sole diagnosis of non-melanoma skin cancer, and (4) have no known contraindications precluding safe participation in non-medically supervised PA per American College of Sports Medicine (ACSM) guidelines and self-report on the Physical Activity Readiness Questionnaire for Everyone (PAR-Q+) [20,21], or receive appropriate medical clearance from a treating physician. Medical contraindications requiring physician clearance included diabetes, renal disease, and atherosclerotic cardiovascular disease [22]. Exclusion criteria for cancer survivors included (1) age less than 18 years, (2) inability to speak and read English, (3) sole diagnosis of non-melanoma skin cancer, and (4) known medical contraindications to non-medically supervised PA without receiving clearance from a treating physician. Cancer survivors were defined as individuals living following a cancer diagnosis [1]. There were no additional eligibility criteria regarding cancer type (except for exclusion based on a sole diagnosis of non-melanoma skin cancer), treatment(s) received, time since diagnosis or treatment, or current cancer- or treatment-related sequelae. Eligibility criteria were broad so that the pilot study would be pragmatic and generalize to the wide range of cancer survivors who might participate in community-based PA programs [23]. Cancer survivors were encouraged, but not required, to invite family or friend partners to participate in the study. These individuals had to meet the same eligibility criteria except for previous cancer diagnoses.

### 2.2. Procedures

Recruitment occurred via email blasts and social media posts targeting members of the Suncoast YMCA, flyers posted in Suncoast YMCA locations, and a newsletter emailed to participants in MCC’s Total Cancer Care (TCC) data repository protocol, which helps connect investigators and consenting patients and survivors to relevant research opportunities. Interested individuals contacted YMCA personnel to provide their personal contact information, and YMCA personnel passed this information along to MCC study personnel. MCC personnel then contacted interested individuals to discuss the study and complete PAR-Q+ screening. Participants whose PAR-Q+ responses required additional medical clearance received instruction to obtain documented physician clearance to participate. All participants provided electronic informed consent via the Research Electronic Data Capture (REDCap) system (REDCap Consortium, Nashville, TN, USA) prior to engaging in any study procedures or providing study data [24]. Eligible cancer survivors were encouraged to provide contact information for family or friend partners who may want to participate with them, and MCC study personnel contacted these individuals for phone screening following the same procedures.

Following informed consent, participants received baseline study questionnaires via email and were scheduled for in-person appointments with an exercise trainer at the Greater Palm Harbor branch of Suncoast YMCA. At this appointment, participants conducted in-person assessments of fitness, physical functioning, and anthropometrics. Participants were encouraged to attend Project Rally sessions from their baseline assessments through the program conclusion in April 2024, with durations of program participation varying based on time of enrollment. Participants completed follow-up questionnaires and in-person assessments within 2 weeks of the program’s conclusion.

### 2.3. Project Rally Program

Project Rally sessions were held for 2 h per day, 5 days per week (i.e., Monday, Wednesday, and Friday evenings from 5 to 7 pm and Tuesday and Thursday mornings from 8 to 10 a.m.). All sessions were held on 3 outdoor pickleball courts at the Greater Palm Harbor YMCA. Because the courts are outdoors and do not have lights, the schedule for evening sessions shifted to 4 to 6 p.m. as sunset occurred earlier in the fall and winter. Participants were encouraged to attend at least 2 h of Project Rally programming each week with no limit to how much programming they could attend. Attendance (including arrival and departure times) was recorded on sign-in sheets at each session. Participants received 3 incentives upon study enrollment: 1 year of YMCA access, a pickleball paddle, and a Project Rally t-shirt.

All Project Rally programming was led and supervised by a YMCA coach holding an exercise training certification from the National Academy of Sports Medicine (Gilbert, AZ, USA) and pickleball coaching certification from USA Pickleball (Scottsdale, AZ, USA) and the Professional Pickleball Registry (Zephyrhills, FL, USA). The coach started each Project Rally session with 10 min of structured warm-up activities, including walking laps around the courts and a combination of dynamic and static stretches. Session activities were tailored specifically to participants’ needs and abilities, including coach-led, small group drills to learn and practice various pickleball shots (i.e., serves, baseline shots, shots near the net known in pickleball as “dinking”, and volleys), court spacing and movement strategies in pairs, and scoring. As players improved in skill and familiarity with pickleball, they graduated to open, “mixer”-style play in which they engaged in game play rotating between different partners and opponents. All game play occurred in the common doubles format. Participants were encouraged to break off into smaller groups to practice “rallying” independently (i.e., returning shots back and forth across the net) and to take breaks when desired. Participants were required to wear appropriate athletic footwear and clothing, and they were encouraged to bring and refill water bottles and to protect their skin from sun exposure. As Project Rally enrollment grew in participants with various abilities, the coach developed a list of standards and guidelines to help ensure smooth operation and maintain positive group dynamics (Appendix A).

### 2.4. Measures

#### 2.4.1. Demographic and Clinical Data

Participants reported demographic and clinical information on a questionnaire at baseline. Data included sex, age, race and ethnicity, marital status, cancer survivor status, and zip code of residence. Cancer survivors also reported the following characteristics: cancer type(s); current statuses of cancer (i.e., living with cancer vs. cancer-free), treatment (i.e., receiving treatment vs. completed treatment), and surveillance (i.e., undergoing routine surveillance vs. no longer undergoing routine surveillance); treatment(s) received; and month and year of last cancer treatment.

#### 2.4.2. Feasibility and Acceptability

Aspects of Project Rally identified for feasibility evaluation included (1) recruitment (i.e., percentage of individuals who enrolled from those who discussed the study with research personnel; target ≥ 50%) [25,26], (2) retention (i.e., percentage of individuals who completed follow-up assessments from those who enrolled; target ≥ 75%) [26], and (3) adherence (i.e., percentage of session hours attended based on the recommendation of 2 h per week; target ≥ 75%) [27]. Weeks with limited programming due to YMCA holiday closures or the YMCA coach being unavailable were factored into the denominator for adherence calculations proportionately. To further inform feasibility evaluation, we ascertained reasons for declining study participation and reasons for not completing the program. To evaluate acceptability, participants completed a questionnaire adapted from the Participant Evaluation of Feasibility and Acceptability at follow up [28]. This questionnaire consisted of 9 Likert-type items (each rated on a 5-point scale from strongly disagree to strongly agree) and 4 open response questions. The 4 open-response questions were as follows: “What aspects of Project Rally were most helpful to keep you participating?”, “What were the biggest challenges or barriers to participating in Project Rally?”, “What, if any, benefits do you feel you received from participating in Project Rally?”, and “What, if any, suggestions do you have to improve Project Rally?”.

#### 2.4.3. Self-Reported Outcomes

To measure PA, we used a modified version of the Godin–Shephard Leisure-Time Exercise Questionnaire (GLTEQ), which has been widely used and validated among cancer survivors [29,30]. Whereas the original GLTEQ ascertains weekly frequencies of mild, moderate, and strenuous intensity aerobic exercise, the modified version also ascertains the average duration of exercise at each intensity. Using this modified version, we multiplied weekly frequency by average duration to compute weekly minutes of mild, moderate, and strenuous aerobic exercise. Weekly minutes of moderate and strenuous-intensity aerobic exercise were added to estimate weekly minutes of moderate-to-strenuous aerobic exercise, which corresponds to MVPA based on the descriptions in the questionnaire. We followed scoring guidelines to compute the Godin “score” (i.e., 9× strenuous frequency + 5× moderate frequency + 3× mild frequency) [30].

Social support for PA was measured using the Physical Activity and Social Support Scale (PASSS), which has demonstrated validity and reliability in adults over a wide age range [31]. The PASSS was selected from among measures of social support for PA for its unique incorporation of subscales evaluating all 5 established forms of functional social support (i.e., emotional, companionship, instrumental, informational, and validation) and for its assessment of social support from anyone (i.e., not limited to friends and family) [31,32]. We anticipated that all 5 forms of functional social support may be important to evaluate in a PA program that involves learning a new sport. Furthermore, we wanted participants to draw from social support they may have received from fellow program participants and the YMCA coach without assuming they would characterize them as friends. PASSS responses were scored following the published protocol to produce the 5 subscale scores (each with a potential range of 0–28) and an overall score (potential range of 0–140); in each, a higher score indicates more social support.

We measured PA enjoyment using the Physical Activity Enjoyment Scale-8 (PACES-8), a modified version of the original PACES that has demonstrated validity and reliability in adults, including older adults [33,34,35]. PACES-8 responses were scored following the published protocol to produce an overall score with a potential range of 7–56, with higher scores indicating more enjoyment.

We measured exercise motivation using the Behavioral Regulation in Exercise Questionnaire-3 (BREQ-3), which has demonstrated validity and reliability in adult populations and has been used in cancer survivorship contexts [36,37,38]. The BREQ-3 consists of 24 items and scores exercise motivation in 6 subscales based on Self Determination Theory and the continuum of self-determined motivation: amotivation, external regulation, introjected regulation, identified regulation, integrated regulation, and intrinsic regulation [39]. BREQ-3 responses were scored following the published protocol to produce the 6 subscale scores (each with a potential range of 0–4), with higher scores indicating greater degrees of each type of behavioral regulation. We also computed the Relative Autonomy Index (RAI), which combines subscale scores in a weighted composite score with a potential range of -19–24. A higher RAI score indicates a greater degree of autonomous motivation for exercise.

We measured stress using the Perceived Stress Scale-10 (PSS-10), a 10-item questionnaire that has demonstrated validity and reliability in adult populations, including cancer survivors [40,41]. PSS-10 responses were scored following the published protocol to produce total scores with potential range 0–40, with higher scores indicating higher perceived stress.

We measured health-related quality of life using the widely validated 36-item Medical Outcomes Short Form (SF-36), which has been used extensively in cancer settings [42,43,44]. SF-36 responses were scored following a published protocol to determine 8 specific subscale scores: physical functioning, emotional well-being, role limitations due to physical health, role limitations due to emotional problems, energy/fatigue, social functioning, pain, and general health. In each subscale, a higher score indicates better quality of life.

We measured self-reported physical functioning using the Patient Reported Outcomes Measurement Information System (PROMIS) Short Form Version 1.0—Physical Function 12a. This PROMIS questionnaire consists of 12 questions about mobility and capabilities regarding exercise, self-care, and daily tasks and has been validated and used extensively in cancer settings [45,46]. PROMIS responses were scored following the published protocol, and raw scores were converted to T-scores (standardized score with mean = 50 and SD = 10) in which a higher score indicates better physical functioning.

#### 2.4.4. Objective Outcomes

We measured body mass index (BMI) using a bioelectrical impedance analysis (BIA) scale (InBody H20N, InBody USA, Cerritos, CA, USA). The BIA scale performed metric conversions for self-reported height (from feet and inches to meters) and weight (from pounds to kg) to provide BMI in kg/m^2^. Body composition variables (i.e., skeletal muscle mass and fat mass in pounds, body fat percentage) were measured using the same BIA scale. Skeletal muscle mass and fat mass were converted to kg for data presentation and analysis.

Balance was measured using a unipedal stance test [47], conducted separately for right and left legs while participants were barefoot and with eyes open. Participants were instructed to stand on one leg unassisted and were timed (in seconds) from when the non-testing foot left the ground until it touched the ground, with timing stopped at a maximum of 60 s. The longer balance duration between the 2 limbs was used for data presentation and analysis.

Flexibility in the lower back and hamstrings was measured using a seated sit-and-reach test [48]. Participants were instructed to sit on the floor with their legs outstretched and feet (while barefoot) positioned flat against a sit-and-reach box (Baseline Sit and Reach, Fabrication Enterprises, Elmsford, NY, USA). Keeping their knees flat against the floor, participants reached their fingertips towards the slide on the box and pushed it as far away as possible, holding the end position for 3 s. The recorded distance was the shift in the slide (in cm), with a reach distance of 22.9 cm indicating the position of feet against the box.

Lower limb strength and endurance were measured using a 30 s chair stand test [49]. The test began with participants seated with their arms held across their chest in a standard height chair (17 inches) with a hard seat and no armrests. Following a practice repetition to guide proper form, participants were instructed to complete as many full stands as possible within 30 s, returning to the initial seated position each time and without using their arms. The score was the number of full stands performed in 30 s.

Grip strength (in kg) was measured using a Jamar handgrip dynamometer (Performance Health, Warrenville, IL, USA) [50]. Dynamometer spacing was adjusted according to participant preference at baseline and remained constant at both assessment time points. Participants conducted 3 trials with each hand while seated comfortably and with their elbows bent at 90-degree angles, alternating between hands and following instructions to squeeze with maximal strength for each trial. The maximum force exerted in these 6 trials (regardless of hand) was used for data presentation and analysis.

Upper limb strength and endurance were measured using a 30 s arm curl test [51]. Female participants used a 5-pound dumbbell, and male participants used an 8-pound dumbbell. While seated and keeping their elbow stationary at their sides, participants performed as many complete biceps curls as possible in 30 s. The test was performed separately using each arm, and the higher number of repetitions (regardless of arm) was used for data presentation and analysis.

Dynamic balance was measured using an 8-foot up and go test [51]. Participants started in a seated position in a standard height chair (17 inches) and were instructed to stand, walk to a cone positioned 8 feet from the chair, turn around the cone, and return to a seated position in the chair. The time required to return to a seated position (in seconds) was recorded.

Submaximal aerobic fitness was measured using a 6 min walk test [52]. Participants were instructed to walk as far as possible in 6 min, passing between and turning around 2 cones spaced 30 m apart. Distance walked (in meters) was recorded for analysis.

#### 2.4.5. Data Analyses

We targeted accrual of 25 cancer survivors and up to 25 family or friend partners based on (1) anticipated recruitment success over a 5-month period and (2) capacity to participate comfortably and safely at the single YMCA location. With a single YMCA coach dedicated to leading and supervising Project Rally programming, we anticipated that 25 cancer survivors (and up to 25 family or friend partners) would allow for manageable program delivery with attendance spread across session schedules. Furthermore, we anticipated that 25 cancer survivors would provide sufficient data to estimate the feasibility of recruitment, retention, intervention adherence, and acceptability metrics. The study was not powered to detect statistically significant changes in exploratory outcome measures.

Demographic and clinical variables and feasibility and acceptability metrics were summarized using appropriate descriptive statistics. Open-ended responses from the acceptability questionnaire were analyzed qualitatively by a trained coder following a content analysis strategy to systematically define units of text, group them into meaningful categories, and quantify their occurrence among participants’ responses [53]. Changes in exploratory outcome measures were evaluated using paired *t*-tests or Wilcoxon signed rank tests based on variable distributions, with separate tests performed for cancer survivors, non-cancer survivors, and the combined sample. All statistical analyses were conducted using IBM SPSS Statistics (Version 26).

## 3. Results

### 3.1. Study Sample

**Figure 1:**
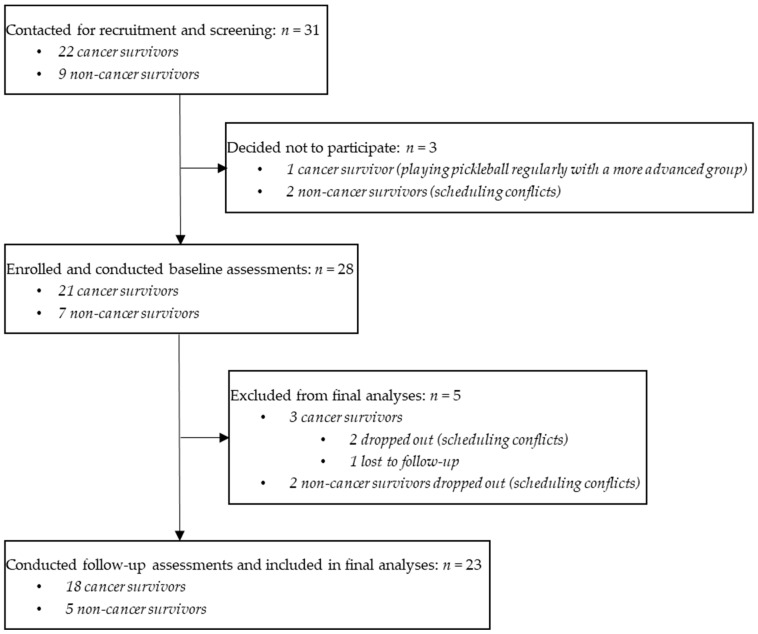
Study flow diagram.

Figure 1 depicts the flow of participants through the study. Cancer survivors expressed interest and were screened for eligibility between September 2023 and 22 January 2024. Twenty-one of these individuals immediately met screening criteria and were offered enrollment. One cancer survivor required and received medical clearance from a treating oncologist to be eligible due to a history of coronary artery disease and chronic kidney disease. Twenty-one of the 22 eligible cancer survivors agreed to participate and provided informed consent. Nine of the 21 cancer survivor participants referred a non-cancer survivor family member or friend to participate with them. All nine non-cancer survivors met screening criteria and were offered enrollment, and seven agreed to participate and provided informed consent. Reasons for electing not to participate included already playing pickleball regularly with a more advanced group (one cancer survivor) and scheduling conflicts (two non-cancer survivors). After enrollment, four participants (two cancer survivors and two non-cancer survivors) developed scheduling conflicts and dropped out of the study, and one cancer survivor was lost to follow-up. A total of 23 participants (18 cancer survivors and 5 non-cancer survivors) completed follow-up assessments and were included in analyses.

Table 1 includes characteristics of cancer survivors and non-cancer survivor participants who completed follow-up assessments. Cancer survivors were mostly female (94.4%), non-Hispanic white (77.8%), married (72.2%), and had a median age of 61.5 years (range 47–76). Breast cancer represented the most frequent diagnosis (55.6%), followed by hematological cancer (22.2%). Two participants had been diagnosed with multiple cancer types. Majorities of cancer survivors underwent surgical tumor resection (72.2%) and chemotherapy (55.6%) prior to study participation, and nearly half (44.4%) underwent radiation therapy. Cancer survivors had a wide range of time between their last cancer treatment and study enrollment (median 30 and range 3–251 months). All cancer survivors were disease-free at study enrollment, with most (83.3%) still undergoing regular disease surveillance. Only two cancer survivors (11.1%) were still receiving cancer treatment at study enrollment; both were receiving hormonal therapy for breast cancer. Non-cancer survivors were all male and mostly non-Hispanic white (80.0%), married (80.0%) with a median age of 58 years (range 54–76). Median estimated driving distances from home to the program site were 4.0 miles (range 2.1–19.8) and 3.2 (range 2.1–11.4) for cancer survivors and non-cancer survivors, respectively. Average duration of program participation was 15.0 weeks (SD = 6.1) and 19.0 weeks (SD = 7.0) for cancer survivors and non-cancer survivors, respectively. Study participants experienced no adverse events related to Project Rally sessions or study assessments.

### 3.2. Feasibility and Acceptability

Table 2 shows study feasibility metrics. Cancer survivor recruitment (*n* = 21) was slightly short of the accrual target of 25 in the 6-month recruitment period, and only 9 cancer survivor participants referred non-cancer survivor family members or friends to participate. However, recruitment rates among those who expressed interest (95.5% among cancer survivors, 77.8% among non-cancer survivors, and 90.3% overall) exceeded the a priori target of 50%. Retention of cancer survivors (85.7%) and overall retention (82.1%) exceeded the a priori target of 75%, but retention of non-cancer survivors (71.4%) was slightly below the target. Among participants who completed the study, rates of adherence (percentage of recommended session hours attended) exceeded the a priori target of 75%, with cancer survivors attending 87.0%, non-cancer survivors attending 78.8%, and both groups combined attending 85.2% of recommended session hours, respectively.

**Figure 2:**
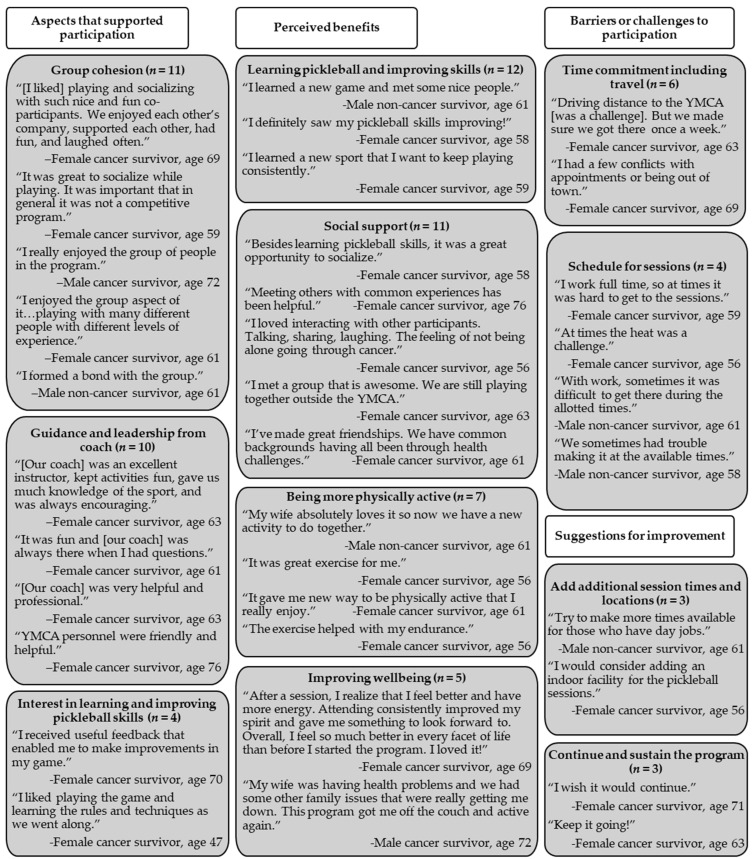
Qualitative acceptability data.

Table 3 shows study acceptability metrics, highlighting strong favorability ratings across all aspects of Project Rally. Average scores on all acceptability items were between 4 (“agree”) and 5 (“strongly agree”). Figure 2 describes the 4 overarching themes and 11 subthemes that emerged from open responses to acceptability questionnaire items, including representative quotes for each subtheme.

### 3.3. Outcomes

Table 4 shows results from self-reported outcome measures. Cancer survivors reported statistically significant increases in weekly moderate-to-strenuous PA (mean increase ± SD = 82.2 ± 152.2 min, *p* = 0.045), strenuous PA (49.7 ± 76.0 min, *p* = 0.030), and overall Godin score (11.9 ± 18.1 points, *p* = 0.020) from baseline to follow-up. Though non-cancer survivors reported no significant changes in PA, the same improvements were statistically significant with all participants combined (75.6 ± 144.2 min, *p* = 0.038; 44.5 ± 84.7 min, *p* = 0.049; and 11.2 ± 17.3 points, *p* = 0.012, respectively). Cancer survivors reported significant increases in companionship social support for PA (4.2 ± 6.2 points, *p* = 0.020). The increase in companionship social support for PA was also statistically significant with all participants combined (3.9 ± 7.1 points, *p* = 0.035). There were no significant changes in PA enjoyment, exercise motivation, perceived stress, multidimensional social support, health-related quality of life, or self-reported physical functioning among cancer survivors, non-cancer survivors, or with all participants combined (all *p* > 0.050).

Table 5 shows results from objective outcome measures. Cancer survivors demonstrated statistically significant increases in 30 s chair stands (2.1 ± 2.5 repetitions, *p* = 0.006) and 30 s arm curls (2.7 ± 3.2 repetitions, *p* = 0.003). The same functional improvements were statistically significant with all participants combined (2.2 ± 3.1 repetitions, *p* = 0.006; and 2.3 ± 3.1 repetitions, *p* = 0.003, respectively). Including all participants, there was a small but statistically significant increase in BMI (0.3 ± 0.6 kg^2^/m^2^, *p* = 0.031), and there were improvements in the 8-foot up and go test and 6 min walk test (0.6 ± 0.9 s, *p* = 0.006; and 52.2 ± 65.9 m, *p* = 0.045, respectively). 

## 4. Discussion

This study’s findings support the feasibility and acceptability of delivering the Project Rally pickleball program for cancer survivors in a community-based setting and highlight important lessons to guide design and delivery of future programming. Preliminary outcomes suggest that participating in Project Rally may help cancer survivors increase PA, experience more social support for PA, and improve important aspects of physical functioning and fitness.

The pilot study established the feasibility of recruitment, retention, and adherence in Project Rally with rates surpassing a priori targets. Recruitment rates were high (95.5% among cancer survivors, 77.8% among non-cancer survivors, and 90.3% overall) and exceeded our 50% target, suggesting that the program was appealing. The 50% recruitment target was based on results from two previous, larger programs including PA or exercise training for cancer survivors: Active Living After Cancer (45% enrollment rate) and FitSteps for Life (42% enrollment rate) [25,26]. In a recent systematic review by Reynolds et al., the median recruitment rate across 86 exercise trials for cancer survivors was 38% [54]. Rates of retention in Project Rally among cancer survivors (85.7%) and overall (82.1%) exceeded our 75% target, suggesting that participants were willing and able to complete the program and study. The 75% retention target was also based on results in Active Living After Cancer (68% retention in a 3-month intervention) and FitSteps for Life (86% retention after 3 months and 60% retention after 6 months of intervention) [25,26]. A recent systematic review by Hu et al. found a median retention rate of 80.18% across exercise trials (4–24 weeks in duration) involving cohorts with mixed cancer diagnoses [55]. Finally, rates of intervention adherence also exceeded the target (75%), with cancer survivors attending 87.0%, non-cancer survivors attending 78.8%, and the combined sample attending 85.2% of recommended session hours, respectively. The 75% adherence target was based on results from previous exercise interventions for cancer survivors in which participants attended 62–78% of prescribed sessions [27]. Promising adherence rates suggest that participants were motivated to attend sessions and that logistical considerations such as scheduling and transportation did not preclude consistent engagement. Bullard et al. identified an average of 77% aerobic exercise adherence across studies intervening in populations with various chronic health challenges [56]. Though it is difficult to project rates of recruitment, retention, and intervention adherence in a future randomized controlled trial testing the effectiveness of Project Rally for improving cancer survivorship outcomes, pilot findings are promising. Importantly, findings also demonstrated that Project Rally was safe, with participants experiencing no adverse events related to programming or assessments.

Acceptability ratings and qualitative themes complemented feasibility results in demonstrating the promise of Project Rally. Average scores for all 9 acceptability items were between 4 and 5, indicating that participants tended to agree or strongly agree with statements indicating that they enjoyed the program, that design and instruction fit their needs, and that they benefited from participating. When asked to describe aspects of the program that supported participation, participants largely highlighted group cohesion and the social nature of Project Rally sessions, and they noted the positive influence of the YMCA coach through provision of encouragement and helpful instruction. These themes overlapped with a third theme regarding support for participation, which was that participants were motivated to learn to play pickleball and improve their skills. When asked about barriers or challenges to participation, participants highlighted the time commitment (including travel time) and scheduling aspects (i.e., conflicting commitments and heat). Participants cited numerous perceived benefits from participating in Project Rally, including learning pickleball and improving their skills, making friends and socializing with other cancer survivors, increasing PA, and improving physical and psychosocial wellness. Suggestions for improving Project Rally included improving accessibility (i.e., timing and location of sessions) and finding ways to continue and sustain the program. These acceptability findings provide an important framework to build upon and improve Project Rally delivery, such as adding locations and session times to improve accessibility and developing formal training for coaches that includes the standards and operating guidelines our YMCA coach successfully implemented in the pilot study (Appendix A).

Despite promising recruitment, retention, adherence, and acceptability, total enrollment of cancer survivors in the Project Rally pilot study fell short (*n* = 21, or 84% of the target *n* = 25). We attribute this to a few potential reasons. First, due to personnel limitations, we used only passive recruitment strategies. Recruitment was limited to posting flyers and emailing listservs of YMCA of the Suncoast members and former Moffitt research participants, requiring that interested individuals contact YMCA or Moffitt research personnel to initiate further recruitment and screening. Second, programming was available at a single YMCA within the overlapping but large catchment areas of the YMCA of the Suncoast and the Moffitt. The single center offering likely limited programming accessibility to a small percentage of cancer survivors who may have been interested. YMCA of the Suncoast consists of 7 total YMCAs, and additional “branches” within the Moffitt Catchment Area (Tampa Metropolitan Area YMCA and YMCA of Greater St. Petersburg) consist of 13 and 3 YMCAs, respectively. These sites provide clear opportunities to expand program offerings and increase accessibility for a large population of Tampa Bay area cancer survivors. As offerings increase and cover larger geographic areas, we will utilize more active recruitment strategies, such as screening Moffitt clinics and querying electronic medical records for potentially eligible cancer survivors whose home addresses increase the likelihood of feasible participation.

Only nine cancer survivor participants referred non-cancer survivor family members or friends to study personnel for recruitment, resulting in seven non-cancer survivor participants enrolling in the study. This suggests that most cancer survivor participants may have viewed Project Rally as an opportunity to develop new social connections with others facing similar circumstances or pursuing similar goals in cancer survivorship. Qualitative data from cancer survivor participants support this explanation, with 11 highlighting making new friends in a supportive group as a key benefit. Though non-cancer survivors comprised a smaller portion of the pilot study sample and the program may have fulfilled different needs for them, it is important to note that they also rated Project Rally favorably.

Though this pilot study was not designed or powered to measure the effectiveness of Project Rally in improving outcomes, preliminary findings were promising. On average, the pilot sample reported already meeting the aerobic PA recommendation for post-treatment cancer survivors (≥ 150 min of moderate-to-strenuous PA per week) at baseline, but Project Rally participation still led to substantial and statistically significant increases in strenuous and moderate-to-strenuous PA. Participants also reported a statistically significant increase in companionship social support for PA, aligning with the benefits and supports regarding group cohesion they noted in responses to acceptability prompts. Finally, participants demonstrated statistically significant improvements in different aspects of physical functioning and fitness including improvements in 30 s chair stand repetitions (mean 2.1), 30 s arm curl repetitions (mean 2.7), 8-foot up and go speed (mean 0.6 s), and 6 min walk test distance (mean 52.2 m). To our knowledge, there are no established minimal clinically important differences (MCIDs) for the 30 s arm curl or 8-foot up and go tests, but average improvements in the 30 s chair stand and 6 min walk tests exceeded published MCIDs for clinical populations (two repetitions and 30 m, respectively) [57,58]. Taken together, our findings suggest that Project Rally can help increase PA and improve muscular endurance, aerobic fitness, and dynamic balance among cancer survivors. These are critical improvements in that they mitigate risk for chronic health conditions and help maintain functional independence.

Pilot participants showed no statistically significant changes in PA enjoyment, exercise motivation, perceived stress, health-related quality of life, or self-reported physical functioning. These findings may be attributable to the small sample size and self-selection of pilot study participants who generally reported high PA enjoyment, intrinsic exercise motivation, and health-related quality of life, low stress, and few physical functioning limitations at baseline. High baseline PA and recruitment from among existing YMCA members may have limited potential psychosocial benefits in this pilot study, as participants were likely already seeking and realizing these benefits. There was a small, statistically significant increase in BMI across all participants (0.3 kg/m^2^), but there were no significant changes in skeletal muscle mass, fat mass, or body fat percentage. It is important to note that Project Rally involved no nutritional or weight loss components, and factors such as time of day, hydration status, and clothing were not controlled and may have affected pre- and post-program anthropometric and body composition assessments. Though the directions of mean changes in additional physical functioning outcomes (i.e., balance, flexibility, and grip strength) were generally favorable, the small sample size and the enrollment of participants who were previously active and generally fit likely limited the detection of improvements.

This pilot study had notable limitations. The small sample limits generalizability to a broader, more racially and ethnically diverse population of cancer survivors. It will be important to increase programming accessibility and enroll a more diverse and representative sample in future research. Recruitment strategies and broad eligibility criteria, selected so that the pilot study would be pragmatic and generalize to a wide range of cancer survivors who may participate in community-based PA programs, resulted in a pilot study sample that was generally physically active and with limited apparent sequelae from cancer and cancer treatment before starting the program. Focusing recruitment on or stratifying results in future Project Rally research to highlight cancer survivors who are undergoing treatment or completed treatment more recently, who are completely or insufficiently active, or who are physically deconditioned may increase the magnitude and detectability of physical and psychosocial benefits. Furthermore, this will support the exploration of moderating variables (e.g., PA enjoyment, group cohesion, social support, and program adherence) that may contribute to outcomes. Our pilot study recruitment also resulted in a wide range of program duration, with participants enrolling for a targeted intervention period ranging from 3 to 7 months. PA dose affects physical and psychosocial outcomes, so it will be important to ensure consistency of program duration in future research to improve interpretability.

Our findings make an important contribution to the literature involving PA for cancer survivors by demonstrating the feasibility and acceptability of the Project Rally pickleball program. The novel program design incorporating pickleball as a PA modality for cancer survivors is an important study strength, because it is timely and capitalizes on the sport’s growing popularity and accessibility. We incorporated rigorous strategies to evaluate feasibility and accessibility, providing rich data to build upon in future research. Feasibility and acceptability findings highlight the appeal of pickleball among cancer survivors, for whom implementation of supportive and enjoyable PA programs is a critical need. Furthermore, this pilot study represents a promising research partnership between MCC and the YMCA of the Suncoast. This community partnership and findings regarding feasibility, acceptability, and preliminary outcomes provide an important foundation for future research. There is enormous potential to increase the scope and reach of Project Rally to increase PA and improve wellness among cancer survivors, with YMCAs offering the capability and reliability to deliver and sustain the program.

## 5. Conclusions

This study demonstrates that the Project Rally pickleball program is a feasible and acceptable strategy to increase PA among cancer survivors and may help improve important physical and psychosocial outcomes. Enjoyment, accessibility, and social interaction have contributed to the enormous growth of pickleball, and these characteristics lend promise to increasing and sustaining PA among cancer survivors. Community-based delivery, such as through the YMCA partnership leveraged in this project, was critical for pilot study success and enhances potential to increase reach and sustain programming.

## Figures and Tables

**Table 1:** Participant characteristics.

	Cancer Survivors (*n* = 18)	Non-Cancer Survivors (*n* = 5)
Sex, *n* (%)		
Female	17 (94.4)	0 (0.0)
Male	1 (5.6)	5 (100.0)
Age (years), median (range)	61.5 (47–76)	58 (54–76)
Race and ethnicity, *n* (%)		
Non-Hispanic White	14 (77.8)	4 (80.0)
Hispanic White	2 (11.1)	0 (0.0)
American Indian or Alaskan Native	1 (5.6)	1 (20.0)
Asian	1 (5.6)	0 (0.0)
Marital status, *n* (%)		
Married	13 (72.2)	4 (80.0)
Divorced	4 (22.2)	1 (20.0)
Widowed	1 (5.6)	0 (0.0)
Cancer diagnosis, *n* (%) ^a^		
Breast	10 (55.6)	-
Hematological	4 (22.2)	-
Colorectal	1 (5.6)	-
Appendiceal	1 (5.6)	-
Lung	1 (5.6)	-
Melanoma	1 (5.6)	-
Pancreatic	1 (5.6)	-
Sarcoma	1 (5.6)	-
Thyroid	1 (5.6)	-
Body mass index (BMI; kg/m^2^)		
Median (range)	26.7 (20.0–39.6)	22.5 (21.3–26.9)
18.5 ≤ BMI < 25, *n* (%)	6 (33.3)	3 (60.0)
25 ≤ BMI < 30, *n* (%)	7 (38.9)	2 (20.0)
BMI ≥ 30, *n* (%)	5 (27.8)	0 (0.0)
Cancer treatment(s) received, *n* (%) ^b^		
Surgical tumor resection	13 (72.2)	-
Chemotherapy	10 (55.6)	-
Radiation therapy	8 (44.4)	-
Bone marrow or stem cell transplant	2 (11.1)	-
Hormonal therapy	2 (11.1)	-
Time since last cancer treatment ^c^		
Median months (range)	30 (3–251)	
Less than 1 year, *n* (%)	6 (37.5)	-
1 to 5 years, *n* (%)	5 (31.3)	-
More than 5 years, *n* (%)	5 (31.3)	-
Cancer survivorship status and care during study participation, *n* (%)		
Disease-free and undergoing active surveillance	13 (72.2)	-
Disease-free and undergoing hormonal therapy and active surveillance	2 (11.1)	-
Disease-free and no longer undergoing surveillance	3 (16.7)	-
Estimated driving distance from residence to YMCA location (miles), median (range) ^d^	4.0 (2.1–19.8)	3.2 (2.1–11.4)
Duration of program participation (weeks), mean (SD)	15.0 (6.1)	19.0 (7.0)

^a^ Sum of frequencies exceeds 18 and sum of percentages exceeds 100 due to participants with multiple cancer diagnoses; one participant had breast cancer, a hematological cancer, and melanoma, and one participant had breast cancer and thyroid cancer. ^b^ Sum of frequencies exceeds 18 and sum of percentages exceeds 100 due to participants who received multiple types of cancer treatment. ^c^ Excludes two cancer survivors undergoing hormonal therapy during study participation. ^d^ Based on Google Maps driving distance from residential zip code to YMCA address.

**Table 2:** Feasibility metrics.

Metric	Cancer Survivors	Non-Cancer Survivors	Overall
Recruitment [contacted/enrolled (%)]	21/22 (95.5)	7/9 (77.8)	28/31 (90.3)
Retention [enrolled/completed follow-up assessments (%)]	18/21 (85.7)	5/7 (71.4)	23/28 (82.1)
Adherence ^a^			
Percentage of recommended session hours attended, mean (SD)	87.0 (39.5)	78.8 (46.4)	85.2 (40.1)
Attended 100% of recommended session hours, *n* (%)	6 (33.3)	1 (20.0)	7 (30.4)
Attended at least ≥75% of recommended session hours, *n* (%)	11 (61.1)	2 (40.0)	13 (56.5)
Attended at least ≥50% of recommended session hours, *n* (%)	15 (83.3)	4 (80.0)	19 (82.6)

^a^ Calculated from participants who completed follow-up assessments.

**Table 3:** Acceptability metrics (*n* = 18).

Item	Score, Mean (SD) ^a^
I found the pickleball program fun.	4.6 (1.0)
I was able to participate in the pickleball program without difficulty.	4.5 (1.0)
The pickleball program was worth my time.	4.3 (1.1)
The instruction I received at pickleball sessions was clear.	4.5 (1.0)
I receive the guidance I needed from the pickleball program staff.	4.4 (1.2)
I had or received everything I needed to participate in the pickleball program.	4.7 (1.0)
The intensity of pickleball sessions was appropriate for my ability and needs.	4.6 (1.0)
The frequency of pickleball sessions was appropriate for my ability and needs.	4.6 (0.8)
I was able to make progress (i.e., in pickleball and/or fitness) over the course of the program.	4.5 (0.7)
All items	4.5 (1.0)

^a^ All items scored as 1 = strongly disagree, 2 = disagree, 3 = neutral, 4 = agree, 5 = strongly agree.

**Table 4:** Self-reported outcomes.

	Cancer Survivors (*n* = 16) ^a^			Non-Cancer Survivors (*n* = 3) ^a^		
	Baseline	Follow-Up	*p*	Baseline	Follow-Up	*p*
Physical activity (min/week), mean (SD) ^b^						
Mild	170.3 (181.4)	140.3 (102.7)	0.509	190.0 (75.5)	130.0 (91.7)	0.109
Moderate	102.8 (74.5)	135.3 (92.7)	0.300	160.0 (91.7)	183.3 (95.0)	0.655
Strenuous	59.7 (75.9)	109.4 (76.3)	**0.030**	103.0 (123.6)	120.0 (103.9)	1.000
Moderate-to-strenuous	162.5 (125.0)	244.7 (142.8)	**0.045**	263.0 (146.9)	303.3 (49.3)	0.593
Godin score	34.2 (13.0)	46.1 (17.7)	**0.020**	43.7 (24.1)	51.0 (9.5)	0.593
Social support for physical activity, mean (SD) ^b^						
Emotional	20.0 (7.4)	22.8 (5.7)	0.258	18.0 (9.5)	14.3 (11.0)	0.593
Informational	14.2 (6.1)	16.8 (4.3)	0.114	12.7 (1.5)	10.7 (2.9)	0.285
Instrumental	12.7 (8.6)	12.5 (10.0)	0.779	11.3 (6.7)	2.7 (4.6)	0.109
Validation	9.4 (5.6)	10.3 (3.6)	0.504	10.7 (5.9)	10.0 (2.6)	0.785
Companionship	14.6 (6.9)	18.8 (6.2)	**0.020**	13.7 (5.8)	16.3 (8.1)	0.593
Overall score	70.9 (24.1)	81.1 (22.1)	0.187	66.3 (23.7)	55.0 (23.1)	0.593
Physical activity enjoyment, mean (SD) ^c^	41.7 (7.6)	43.2 (5.4)	0.428	38.7 (4.0)	41.3 (4.2)	0.287
Exercise motivation, mean (SD) ^b^						
Amotivation	0.02 (0.06)	0.00 (0.00)	0.317	0.4 (0.7)	0.3 (0.6)	0.317
External regulation	0.5 (0.6)	0.5 (0.6)	0.952	0.3 (0.6)	0.4 (0.7)	0.317
Introjected regulation	2.1 (1.0)	2.1 (0.9)	0.815	1.4 (0.9)	1.5 (0.5)	0.655
Identified regulation	3.4 (0.5)	3.6 (0.4)	0.254	3.1 (0.9)	3.3 (1.1)	0.157
Integrated regulation	3.0 (0.9)	3.3 (0.7)	0.167	3.1 (1.2)	3.3 (1.1)	0.157
Intrinsic regulation	3.2 (0.7)	3.4 (0.7)	0.294	3.0 (0.7)	2.7 (0.7)	0.180
Relative autonomy index	15.8 (4.2)	17.1 (4.0)	0.118	14.9 (7.6)	14.5 (7.9)	0.180
Perceived stress, mean (SD) ^c^	19.1 (2.2)	18.8 (2.9)	0.371	17.0 (4.0)	19.7 (0.6)	0.319
Health-related quality of life, mean (SD) ^c^						
Physical functioning	95.7 (6.2)	95.3 (5.6)	0.793	93.3 (7.6)	93.3 (7.6)	-
Emotional well-being	78.9 (12.6)	80.8 (11.8)	0.334	81.3 (12.2)	82.7 (8.3)	0.742
Role limitations due to physical health	88.3 (16.0)	80.0 (28.7)	0.371	91.7 (14.4)	91.7 (14.4)	1.000
Role limitations due to emotional problems	82.2 (27.8)	86.7 (21.1)	0.634	100.0 (0.0)	100.0 (0.0)	-
Energy/fatigue	64.7 (20.8)	64.0 (15.8)	0.894	68.3 (10.4)	73.3 (7.6)	0.423
Social functioning	90.8 (12.9)	89.2 (14.1)	0.685	95.8 (7.2)	100.0 (0.0)	0.423
Pain	84.3 (11.5)	84.7 (17.3)	0.946	79.2 (11.3)	60.0 (6.6)	0.202
General health	72.3 (19.4)	73.3 (17.3)	0.670	76.7 (5.8)	75.0 (5.0)	0.423
Physical functioning, mean (SD) ^c^	53.0 (6.0)	51.9 (6.5)	0.453	59.3 (6.9)	56.9 (8.7)	0.184

Bold *p* values indicate statistical significance (*p* < 0.05). ^a^ Numbers of included cancer survivors and non-cancer survivors differ from the study sample (*n* = 18 and *n* = 5, respectively) because participants were considered to have completed the study and were included in analyses if they completed any follow-up assessments; two cancer survivors and two non-cancer survivors who completed objective assessments (presented in Table 5) did not complete self-reported assessments. ^b^ Wilcoxon signed rank tests. ^c^ Paired *t*-tests.

**Table 5:** Objective outcomes.

	Cancer Survivors (*n* = 16) ^a^			Non-Cancer Survivors (*n* = 5)		
	Baseline	Follow-Up	*p*	Baseline	Follow-Up	*p*
Body mass index (kg/m^2^), mean (SD)	27.2 (4.8)	27.5 (4.5)	0.059	23.9 (2.6)	24.2 (2.5)	0.340
Skeletal muscle mass (kg), mean (SD)	26.7 (5.8)	27.0 (5.5)	0.182	35.5 (4.2)	36.4 (4.7)	0.197
Fat mass (kg), mean (SD)	25.0 (6.8)	25.1 (7.4)	0.741	13.4 (8.1)	13.1 (6.9)	0.715
Body fat percentage, mean (SD)	34.6 (7.2)	34.5 (7.7)	0.735	17.0 (8.1)	16.5 (6.5)	0.666
Balance (seconds), mean (SD) ^b^	41.5 (22.6)	41.4 (17.9)	0.983	40.8 (27.0)	49.0 (16.8)	0.436
Flexibility (cm), mean (SD)	15.8 (3.4)	16.3 (3.4)	0.052	18.0 (2.4)	15.8 (7.1)	0.378
Chair stands (repetitions), mean (SD)	16.5 (5.5)	18.6 (4.5)	**0.006**	20.8 (6.0)	23.0 (8.2)	0.365
Grip strength (kg), mean (SD) ^b^	31.8 (9.2)	33.3 (8.7)	0.117	46.4 (11.8)	46.1 (10.6)	0.772
Arm curls (repetitions), mean (SD) ^b^	24.4 (3.5)	27.2 (4.6)	**0.003**	30.8 (3.3)	31.4 (4.9)	0.573
8-foot up and go speed (seconds), mean (SD)	5.2 (0.9)	4.7 (0.9)	0.060	5.2 (1.1)	4.1 (1.1)	0.052
6 min walk test distance (m), mean (SD)	549.6 (71.8)	609.4 (55.0)	0.098	583.7 (114.2)	620.7 (76.3)	0.413

Bold *p* values indicate statistical significance (*p* < 0.05). ^a^ Number of included cancer survivors differs from the study sample (*n* = 18) because participants were considered to have completed the study and were included in analyses if they completed any follow-up assessments; two cancer survivors who completed self-reported assessments (presented in Table 4) did not complete objective assessments. ^b^ Higher value from the two limbs at each time point was used for analyses.

## Data Availability

The data presented in this study are available upon request from the corresponding author.

## Appendix A. Standards and Operational Guidelines for Project Rally Participants Developed by the YMCA Coach

**Standards**	**Rationale and Operational Guidelines**
Be punctual.	Try your best to arrive on time for sessions and warm up with the group. Respect session start and end times to ensure a smooth flow of play for other participants and players who use the courts after sessions.
Be courteous.	Allow other players to warm up or finish their game if courts are being shared. Be patient and wait your turn without interrupting ongoing play. Use down time to stay hydrated and stay “loose”, such as by stretching or walking laps around the outside of the courts.
Use effective communication.	Use clear verbal cues with partners such as calling out “mine” or “yours”, to avoid confusion or collisions when going for a shot. Communicate respectfully with partners and opponents, maintaining a friendly atmosphere and good sportsmanship.
Respect personal space.	Avoid physical contact and give other players enough room to make their shots comfortably. Avoid being a “ball hog” so that your partner has opportunities to practice and develop their game, regardless of their experience or skill level.
Maintain a reasonable noise level.	Avoid loud or distracting noises that could disrupt other players’ focus.
Retrieve stray balls.	Pause game play and promptly pick up and return balls that roll onto your court from other courts. Warn players on other courts when your ball rolls onto their court to maintain safety.
Stay within court boundaries.	Avoid chasing balls that go out of bounds onto neighboring courts or into areas where other activities are taking place.
Control your temper.	Remain calm and composed, even in competitive situations. Avoid displaying anger or frustration and treat fellow players with respect, regardless of the outcomes of points or games.
Offer friendly assistance.	Provide friendly advice or guidance to fellow players, especially those new to the game, but make sure the recipient desires this feedback before providing it. This helps create a supportive environment and encourages skill development.
Maintain a clean environment.	Take responsibility for your belongings and clean up any trash or equipment you have used. Leave the court area clean and organized for the next players.
